# Optimization of Multivalent Gold Nanoparticle Vaccines Eliciting Humoral and Cellular Immunity in an *In Vivo* Model of Enterohemorrhagic Escherichia coli O157:H7 Colonization

**DOI:** 10.1128/msphere.00934-21

**Published:** 2022-01-19

**Authors:** Javier I. Sanchez-Villamil, Daniel Tapia, Alfredo G. Torres

**Affiliations:** a Department of Microbiology and Immunology, University of Texas Medical Branch, Galveston, Texas, USA; b Department of Pathology, University of Texas Medical Branch, Galveston, Texas, USA; University of Florida

**Keywords:** enterohemorrhagic *Escherichia coli*, vaccine, immune response, protection, *E*. *coli* O157:H7, nanovaccine

## Abstract

Enterohemorrhagic Escherichia coli (EHEC) O157:H7 remains a pathogen of significance and high consequence around the world. This outcome is due in part to the high economic impact associated with massive, contaminated product recalls, prevalence of the pathogen in carrier reservoirs, disease sequelae, and mortality associated with several outbreaks worldwide. Furthermore, the contraindication of antibiotic use for the treatment of EHEC-related infections makes this pathogen a primary candidate for the development of effective prophylactic vaccines. However, no vaccines are approved for human use, and many have failed to provide a high degree of efficacy or broad protection, thereby opening an avenue for the use of new technologies to produce a safe, effective, and protective vaccine. Building on our previous studies using reverse vaccinology-predicted antigens, we refine a formulation, evaluate new immunogenic antigens, and further expand our understanding about the mechanism of EHEC vaccine-mediated protection. In the current study, we exploit the use of the nanotechnology platform based on gold nanoparticles (AuNP), which can act as a scaffold for the delivery of various antigens. Our results demonstrate that a refined vaccine formulation incorporating EHEC antigen LomW, EscC, LpfA1, or LpfA2 and delivered using AuNPs can elicit robust antigen-specific cellular and humoral responses associated with reduced EHEC colonization *in vivo*. Furthermore, our *in vitro* mechanistic studies further support that antibody-mediated protection is primarily driven by inhibition of bacterial adherence onto intestinal epithelial cells and by promotion of macrophage uptake and killing.

**IMPORTANCE** Enterohemorrhagic E. coli O157:H7 remains an important human pathogen that does not have an effective and safe vaccine available. We have made outstanding progress in the identification of novel protective antigens that have been incorporated into the gold nanoparticle platform and used as vaccines. In this study, we have refined our vaccine formulations to incorporate multiple antigens and further define the mechanism of antibody-mediated protection, including one vaccine that promotes macrophage uptake. We further define the cell-mediated responses elicited at the mucosal surface by our nanovaccine formulations, another key immune mechanism linked to protection.

## INTRODUCTION

Enterohemorrhagic Escherichia coli (EHEC) is the causative agent of many diarrheal outbreaks around the world, affecting primarily middle- and high-income countries (1, 2). EHEC belongs to the group of Shiga toxin-producing E. coli (STEC) strains, which are associated with disease linked with consumption of contaminated food products, mainly ground beef or produce (3). The most common STEC serotype associated with large outbreaks is O157:H7, which presents a broad spectrum of symptomatology, ranging from mild to severe hemorrhagic diarrhea to the potential development of the life-threatening hemolytic-uremic syndrome (HUS) (3, 4). Shiga toxin (Stx) belongs to a family of AB_5_ toxins classified as Stx1 and Stx2, and although both toxins could be present in EHEC O157:H7 isolates, Stx2 is often associated with severe disease and development of renal failure (5). EHEC O157:H7 belongs to the group of pathogens that possess the locus of enterocyte effacement (LEE), which is responsible for the formation of attaching and effacing (A/E) lesions on epithelial cells (6). Moreover, antibiotic use for the treatment of EHEC-associated infections is contraindicated because it can initiate an SOS response, leading to exacerbation of Stx production and worsening of the disease prognosis (7, 8). Further, EHEC O157:H7 virulence is increased by the presence of a wide variety of adhesion molecules implicated in colonization of the intestine (9, 10). In addition to the main adhesin, intimin, which is the hallmark factor associated with intimate attachment to the intestinal epithelial cells, some other adhesins include fimbriae and pili (6, 11). We have previously demonstrated that EHEC carries 2 operons (*lpf1* and *lpf2*) encoding long polar fimbriae (Lpf), which are critical for EHEC O157:H7 colonization of the colon (10–14). The genes *lpfA1* and *lpfA2* encode the Lpf adhesin proteins LpfA1 and LpfA2, respectively, which are highly prevalent among STEC O157:H7 strains associated with severe or epidemic disease (15, 16). The presence of many virulence factors in the genome of EHEC O157:H7 has hampered the identification of critical immunogenic antigens ideal for the development of an effective prophylactic or therapeutic vaccine (17–19).

Previous approaches to develop an effective vaccine against EHEC O157:H7 infections have exploited several novel and traditional methods, using chimeric proteins, virus-like particles, ghost bacterial cells, or conjugate vaccines (18, 19). However, although some have been tested in clinical studies, to date none have been licensed for human use. With the advent of whole-genome sequencing and advances in bioinformatics, the vaccinology field has advanced to identify ideal antigens. Approaches like reverse vaccinology are primarily based on the scanning of an annotated genome from a pathogen of interest using bioinformatic prediction tools to identify immunogenic antigens, and this novel technology has enabled the vaccinology field to increase the breath of promising candidates and advance in the development of safe and broadly protective vaccines against several bacterial pathogens (20–22). Significant efforts have been made to identify effective vaccine candidates against EHEC O157:H7 and other diarrheagenic E. coli pathotypes, although none are licensed for human use. Moreover, some shortcomings of recent attempts have been the inability to show significant protection in an *in vivo* model and limited use of EHEC-specific antigens that can provide broad protection. Using a bio- and immunoinformatic analysis (17, 23, 24), we have previously identified several EHEC O157:H7-specific surface-exposed antigens containing several B- and T-cell-predicted epitopes, which were selected as top-ranked predicted immunogens. We have also shown that two of these antigens, LomW and EscC, while conjugated to AuNPs, displayed a degree of protection and reduced intestinal colonization associated with an elevated serum and fecal IgG and also secretory IgA responses (25). Most vaccines tested in animal models of EHEC O157:H7 have a common efficacy outcome showing elicited humoral responses that were both systemic and at the mucosal interface and mainly IgG and IgA. However, the cell-mediated protective responses elicited by these vaccines are undetermined. Therefore, we aimed to increase the breadth of protection against *in vivo* colonization by evaluating several surface-encoded EHEC antigens. Those antigens include EscC, a type III secretion system (T3SS) structural protein; LomW, a phage protein; and two fimbrial proteins, LpfA1 and LpfA2, which are well-known EHEC colonization factors (17, 24) and important virulence factors associated with intestinal colonization (10, 14). Our overall goal is to test and refine a vaccination platform highly effective against EHEC O157:H7, using gold nanoparticles (AuNPs) as carriers with a multiantigen display. This platform traditionally has been used in the context of imaging and carrying chemical compounds. However, recent studies from our group and others have exploited its use for a multiantigen display and delivery to elicit robust immune responses (25–30). These particles are inert molecules incapable of inducing an immune response against themselves but offer a rigid surface capable of attaching a wide range of biomolecules to be presented to immune cells. Furthermore, AuNPs have also been found to directly travel across lymphatic vessels to form antigen depot sites or sustain antigen at the site of injection at important lymphatic organs. Therefore, AuNPs have been shown to act as a molecular antigen delivery system, thereby eliciting protective immunity (29–31).

In the present study, we further refine a vaccine formulation, using our previously predicted set of antigens (novel or previously evaluated), use AuNPs as the platform for delivery, and test their protective efficacy against EHEC O157:H7 colonization. Furthermore, we aimed at understanding the protective response at the mucosal interface that may provide protection against EHEC O157:H7. We hypothesized that by incorporating two additional virulence factors involved in initial bacterial recognition of the epithelium, we would broaden the protective response elicited by vaccination. Using a mucosal route of vaccination, we could analyze the protective humoral and cellular responses elicited and determine important correlates of vaccine-induced protection. Together, our mechanistic studies of antibody-mediated protection offer a new perspective on the use of AuNPs to elicit robust tissue-specific and systemic immune responses and warrant further investigation in their use against other complex pathogens. These advancements warrant further investigation in preclinical and clinical human studies.

## RESULTS

### Strategy and synthesis of AuNP vaccine candidates against EHEC O157:H7 colonization.

To increase the breath of the protective responses against EHEC O157:H7 colonization and to analyze the immune protective mechanism, we evaluated four predicted antigens previously identified by our bio- and immunoinformatic analysis, coupling them to our safe AuNP vaccine platform. Two new fimbrial antigen proteins, namely, LpfA1 and LpfA2, together with our previously tested antigens, the phage-encoded outer membrane protein LomW and the type 3 secretion structural protein EscC, were recombinantly expressed and purified using cobalt affinity chromatography. These proteins were tested for endotoxin levels and contained undetectable levels with a limit of detection (LOD) of 0.1 EU/ml. The histidine (His)-tagged proteins were probed using an antihistidine antibody and visualized by Western blotting (see Fig. S1A in the supplemental material). To use these four proteins as a nanovaccine, we covalently coupled them onto the surface of bare spherical 15-nm gold nanoparticles, synthesized by the Turkevich method (32) (Fig. S1B), and assessed for efficient conjugation by UV-visible (UV-Vis) spectroscopy (Fig. S1C). The shift in spectral light within 2 to 4 nm after linker coupling suggests efficient conjugation of proteins onto the surface of AuNPs (Fig. S1C and D). We have consistently demonstrated our capacity to synthesize different EHEC antigens coupled to AuNP for vaccination.

10.1128/msphere.00934-21.1 FIG S1 Synthesis of the components of the AuNP vaccine platform. (A) Western blot analysis with an antibody anti-His showing the purity of LpfA1 (18 kDa), LpfA2 (20 kDa), LomW (28 kDa), and EscC (56 kDa). Purification of His-tagged recombinant protein from E. coli BL21 was performed using Co^2+^ affinity chromatography. (B) Transmission electron microscopy image representing bare AuNPs showing consistency in size and shape. Bar, 50 nm. (C) UV-Vis spectroscopy summary after protein addition to MHDA-conjugated AuNPs. (D) Conjugation diagram representing the conjugation strategy using 16-mercaptohexadecanoic acid (MHDA) forming a covalent bond between the surface of AuNP and protein. Download FIG S1, TIF file, 0.7 MB. Copyright © 2022 Sanchez-Villamil et al. 2022 Sanchez-Villamil et al. https://creativecommons.org/licenses/by/4.0/ This content is distributed under the terms of the Creative Commons Attribution 4.0 International license.

### Immunization with gold nanoparticle-delivered EHEC antigens reduces *in vivo* colonization.

**FIG 1:**
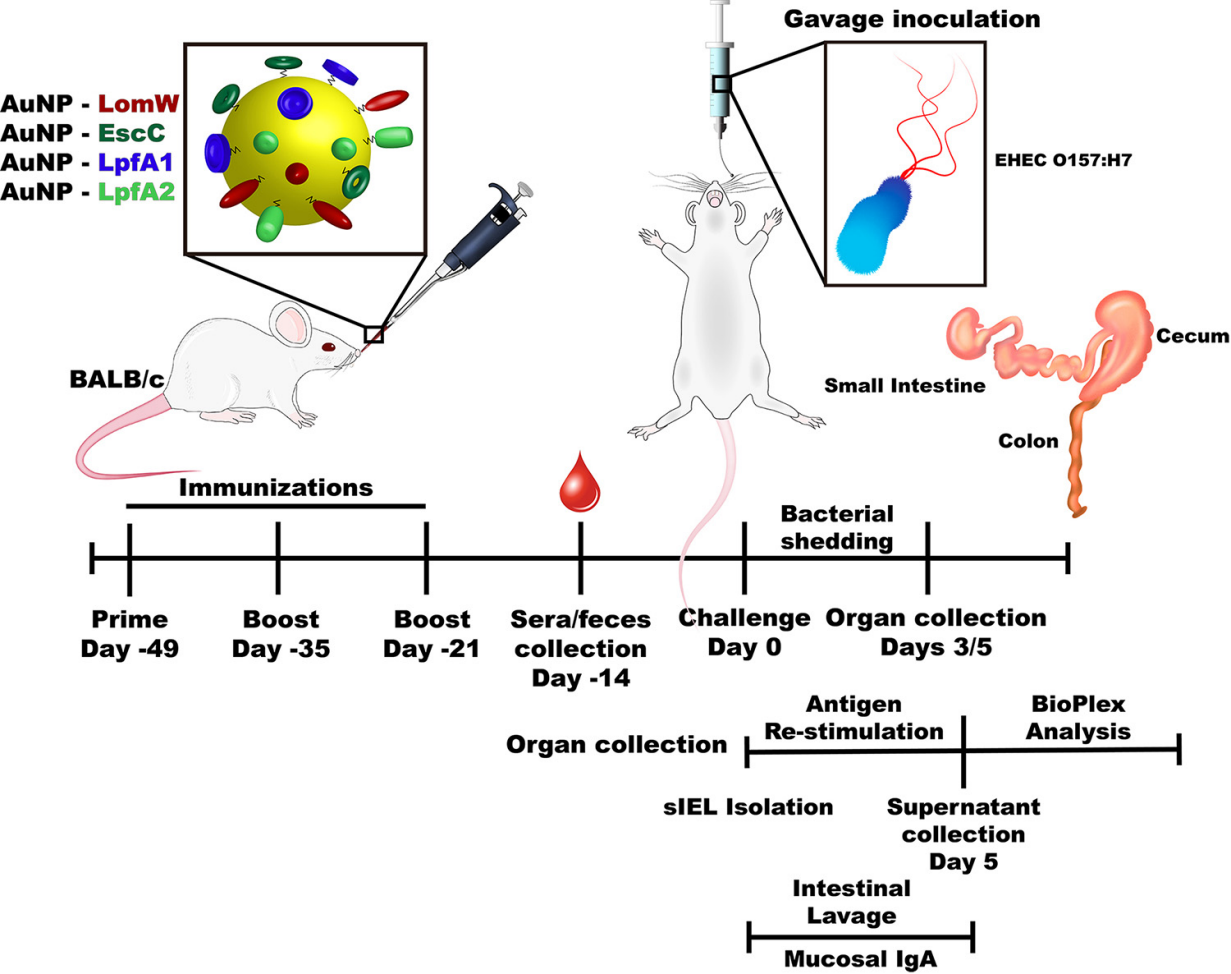
Timeline depicting the intranasal vaccination strategy. Illustration highlights the timeline of the experiments used to test the efficacy and mechanism of protection of the different AuNP protein candidates. BALB/c (*n* = 10) mice were intranasally immunized with three doses of 50-μl formulations in 2-week intervals. Two weeks after receiving the last immunization, serum and fecal samples were obtained to evaluate humoral responses. Seven days after sample collection, animals were challenged with 9 × 10^9^ cells of EHEC O157:H7 via gavage. Individual intestinal segments were collected 3 or 5 days after infection and processed for CFU enumeration. Two weeks after receiving the last immunization, the intestinal epithelial layer (IEL) was isolated and stimulated in the presence of each antigen for 5 days. After stimulation, samples were analyzed by a multiplex cytokine array. Small-intestine lavage samples from immunized and naive animals were obtained 2 weeks after receiving the last immunization to assess secretory IgA levels.

**FIG 2:**
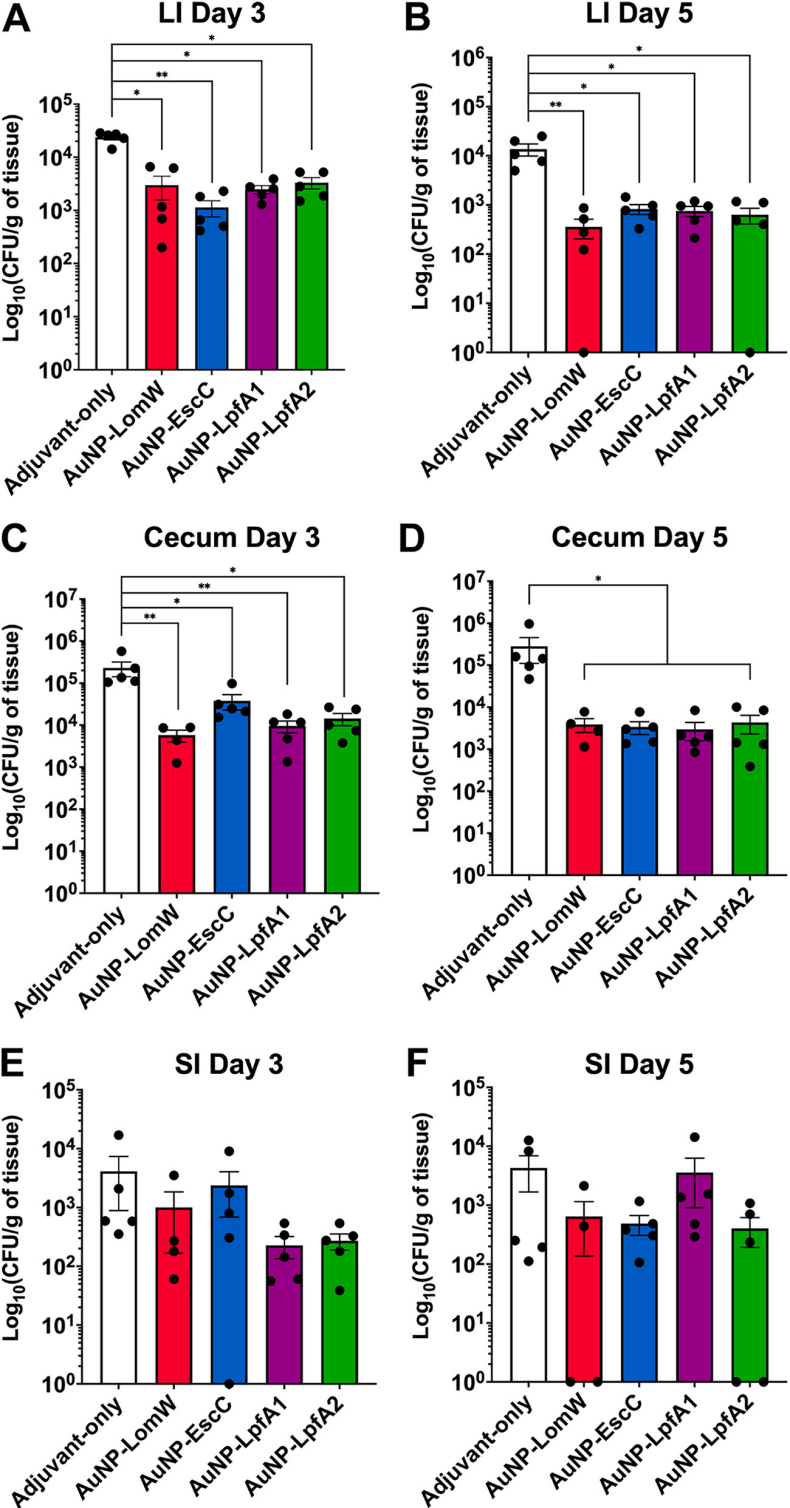
Animals immunized with individual AuNP-coupled vaccines exhibited reduced EHEC colonization. The cecum, small intestine (SI), and large intestine (LI) from vaccinated animals (*n* = 5) were collected three (A, C, and E) and five (B, D, and F) days after EHEC challenge, processed, and plated to assess colonization. Numbers of adhered bacteria were determined per gram of tissue, and adhesion is shown on a log_10_ scale. The bacterial limit of detection (LOD) was 10 CFU/organ. All colonization data are shown as means ± standard errors of the means (SEM) from results determined for each group. Significant differences in organ colonization were determined via one-way ANOVA followed by Tukey’s *post hoc* test (*, *P < *0.05; **, *P < *0.01).

To test the immunogenicity and protective capacity of these four antigens when delivered as conjugates to gold nanoparticles, we immunized BALB/c mice intranasally with 5-μg doses in 2-week intervals (Fig. 1). Following the administration of a prime and two boosts of each vaccine formulation, we assess the *in vivo* protection against EHEC colonization in a mouse model of intestinal colonization. Three weeks after receiving the last immunization (Fig. 1), animals were challenged via gavage with 9 × 10^9^ CFU/mouse of EHEC O157:H7 strain 86-24. Three and five days after challenge, individual segments along the gastrointestinal tract were collected, processed, and plated to enumerate bacterial colonization. Animals immunized with individual formulations, including AuNP-LomW, AuNP-EscC, AuNP-LpfA1, and AuNP-LpfA2, showed significantly lower bacterial colonization along the large intestines (LI) and ceca by days 3 and 5 postchallenge (Fig. 2A to D). However, no significant differences were observed in the small intestine (SI) at any of the time points evaluated (Fig. 2E and F). Taken together, these results demonstrate the ability of the gold nanoparticle EHEC-specific vaccine candidates to reduce colonization when administered intranasally.

### Gold nanovaccine single formulations elicit robust humoral and cell-mediated responses, both systemically and tissue specific, following intranasal immunization.

**FIG 3:**
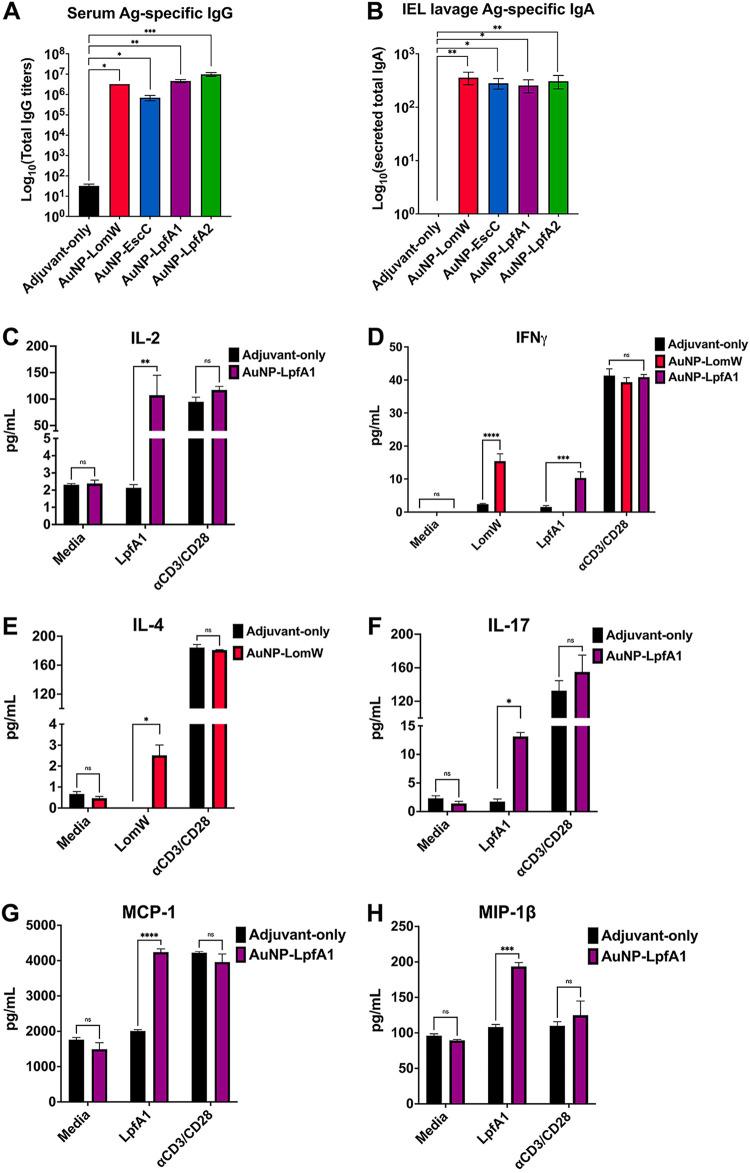
AuNP vaccine intranasal immunization elicits robust antigen-specific humoral and cell-mediated responses associated with reduced bacterial adherence. Systemic and mucosa-specific humoral responses were analyzed using sera (A) and intestinal lavage (B) from immunized animals. The intestinal epithelial layer containing intraepithelial lymphocytes was isolated from immunized animals and stimulated in the presence of each antigen for 5 days. Medium-only and anti-CD3/CD28-coated beads served as negative and positive controls, respectively. (C to H) Supernatants from cells stimulated in the presence or absence of antigen were collected and analyzed for cytokine release using a mouse cytokine Bio-Plex. Only the antigens (LpfA1 and LomW) that showed a cytokine response are shown. All humoral and cytokine data are shown as ±SEM from results determined for each group. Significant differences in humoral or cytokine levels were determined via one-way ANOVA followed by Tukey’s *post hoc* test (*, *P < *0.05; **, *P < *0.01; ***, *P < *0.001).

To evaluate the immunogenicity of the different single-vaccine formulations, we evaluated the antigen-specific humoral responses 2 weeks after receiving the last dose. To assess differences in systemic versus local humoral responses, we collected the serum and intestinal lavage of immunized mice to assess IgG and sIgA antibody responses (Fig. 3). We detected significantly higher antigen-specific IgG and sIgA responses from single-formulation-immunized animals than naive mice (Fig. 3A and B). In addition, we evaluated the cell-mediated tissue-specific responses elicited at the intestinal epithelial layer (IEL). Intraepithelial lymphocytes were isolated from the intestine of immunized animals, and the cell suspensions were restimulated for 5 days in the presence of each antigen to assess antigen recall responses by assessing cytokine secretion. Intraepithelial lymphocytes from AuNP-EscC or AuNP-LpfA2 did not show enhanced cytokine secretion upon restimulation, whereas AuNP-LpfA1-immunized animals in the presence of LpfA1 produced significantly higher levels of interleukin-2 (IL-2), gamma interferon (IFN-γ), IL-17, MCP-1, and MIP-1β than naive controls (Fig. 3C, D, and F to H). Furthermore, animals immunized with the AuNP-LomW formulation elicited higher IFN-γ and IL-4 levels in response to antigen restimulation (Fig. 3D and E). Taken together, these results highlight the ability of single AuNP-protein formulations to elicit robust mixed humoral and tissue-specific cellular responses associated with reduced colonization and protection against *in vivo* infection.

### Immune sera from animals receiving individual AuNP vaccine formulations inhibited bacterial adherence on primary IECs as well as pedestal formation.

**FIG 4:**
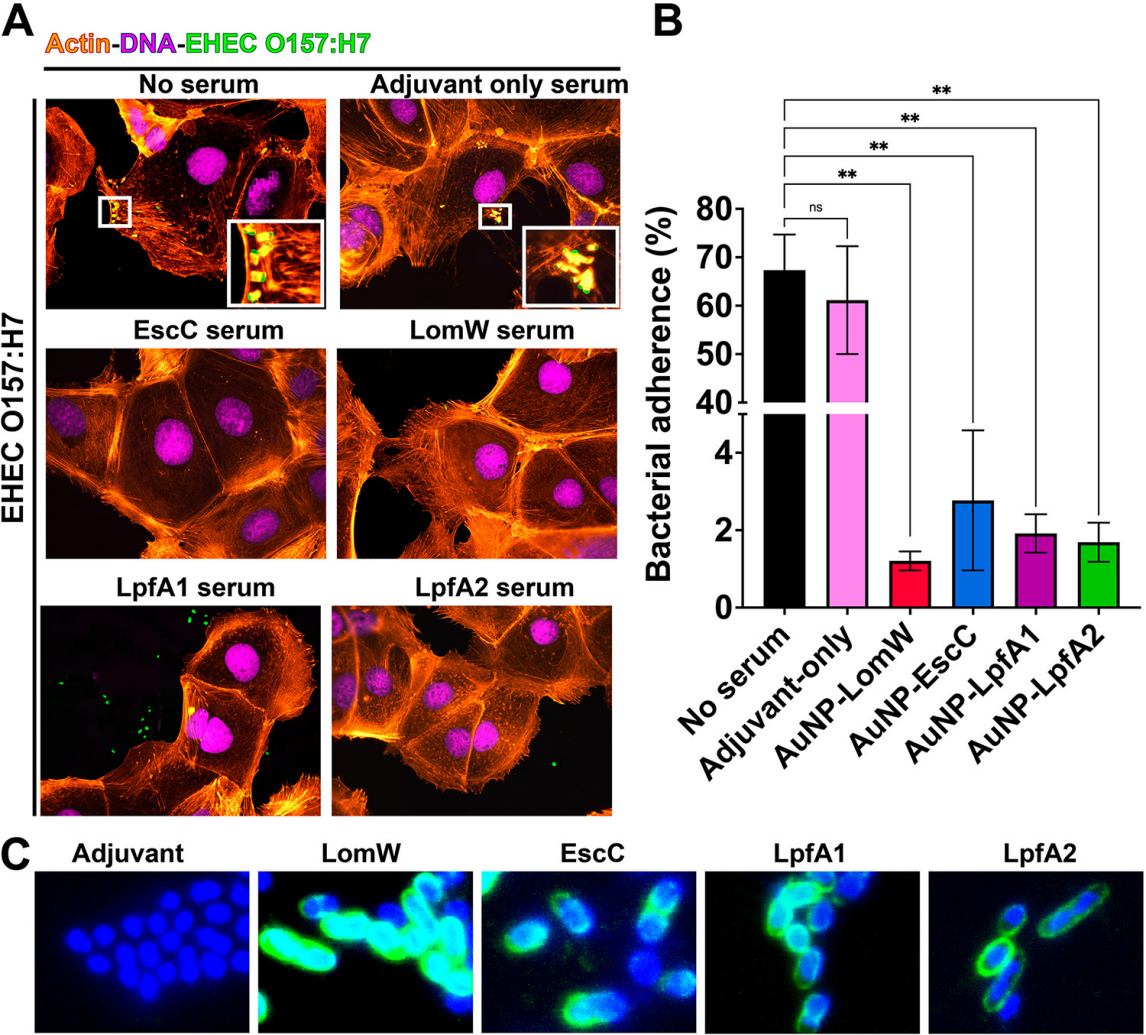
Sera from immunized animals with individual vaccine formulations reduced adherence and pedestal formation on mouse primary intestinal epithelial cells (IECs). (A) Mouse primary IECs were infected at an MOI of 10 with EHEC O157:H7 previously incubated in the presence or absence of naive (adjuvant-only) or immune sera for 1 h at 37°C. After infection, cells were washed to remove nonadherent bacteria and fixed and stained with rhodamine phalloidin and DAPI for IECs and an EHEC-specific primary antibody conjugated to FITC. (B) The percentage of adhered bacteria was calculated 1 h after infection, where cells were washed, detached, and processed for CFU enumeration. (C) Recognition of EHEC O157:H7 by immune sera was visualized using immune or naive sera from vaccinated animals, followed by a secondary goat anti-mouse IgG antibody conjugated to Alexa488. All adherence data are expressed as means ± SEM of results from at least three independent experiments using sera from *n* = 8 mice per group. Significant differences were determined via one-way ANOVA followed by Tukey’s *post hoc* test (**, *P < *0.01; ns, not significant).

To further understand the mechanism of vaccine-induced protection, we evaluated whether serum from immunized mice bind and neutralize bacteria, preventing adherence onto IECs. We observed a complete ablation of EHEC-mediated A/E lesions, as measured by the reduction on pedestal formation, as well as a reduction on adherence onto IECs, of bacteria in contact with immune sera from vaccinated mice compared to untreated or naive sera (Fig. 4A). To quantify the percentage of bacterial adherence onto IECs, a serum inhibition of adherence assay was performed. We detected significantly lower bacterial adherence in the presence of AuNP-LomW, AuNP-EscC, AuNP-LpfA1, and LpfA2 compared to naive or untreated bacteria (Fig. 4B). To assess if the inhibition of adherence was due to the antibody recognition properties from immune serum, immunostaining with the serum from immune animals was used. The sera from immunized animals recognized the outer membrane of EHEC O157:H7 (Fig. 4C), suggesting that serum recognition of surface-exposed antigens is responsible for the inhibition on adherence. Together, these data indicate that AuNP-delivered EHEC-specific vaccine antigens elicit a robust humoral response associated with inhibition of adherence and pedestal formation, a phenomenon mediated by surface-expose antigen recognition.

### AuNP vaccine immunization elicits an antibody-mediated uptake by primary murine macrophages, resulting in bacterial killing.

**FIG 5:**
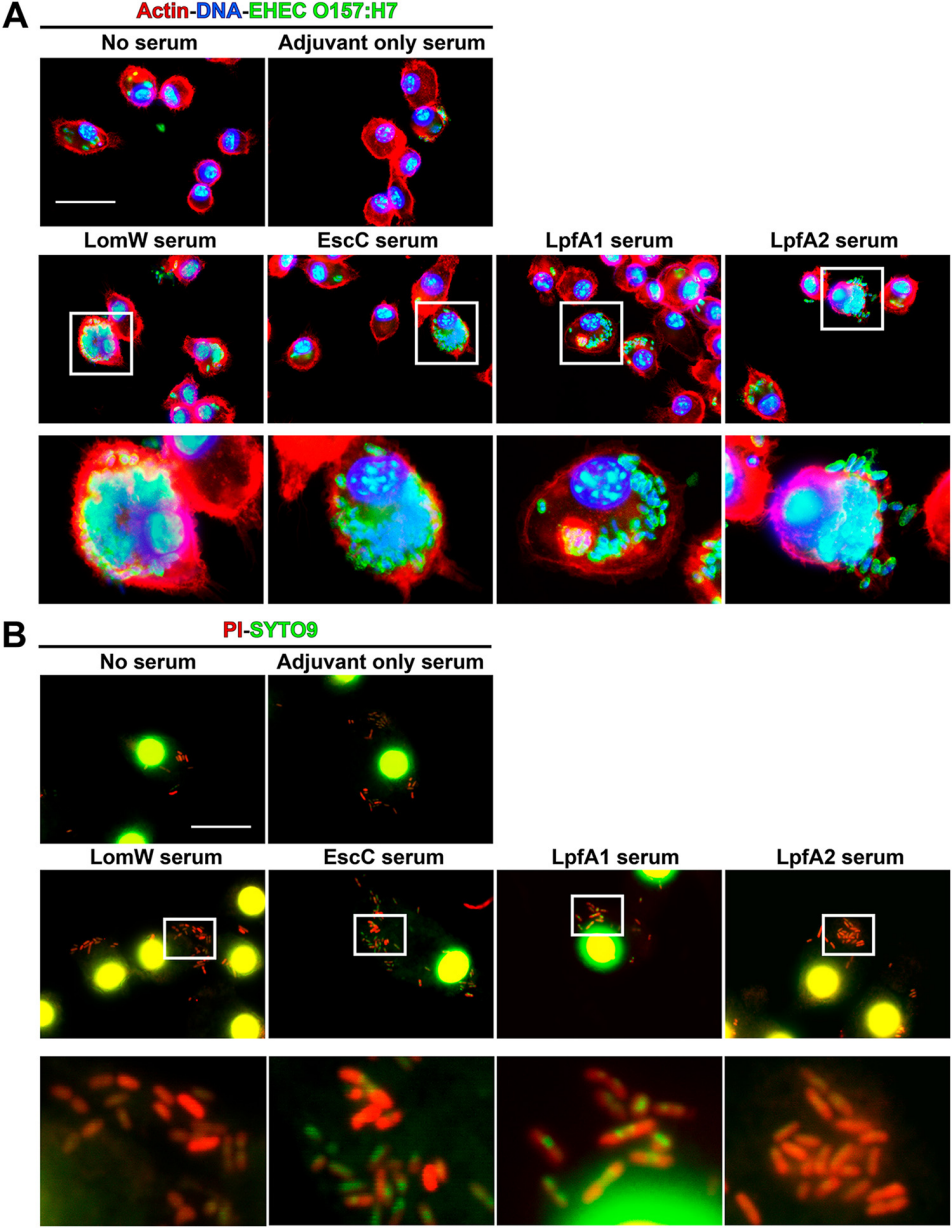
Sera from animals immunized with AuNP vaccines promoted bacterial opsonophagocytosis and killing by mouse primary macrophages. (A) Mouse primary macrophages were infected with bacteria in the presence or absence of immune sera (10% of final volume) from immunized animals with each vaccine formulation (*n* = 5) at an MOI of 10 for 2 h at 37°C. Sera from naive mice (adjuvant only) served as controls. To visualize bacterial uptake by macrophages, 2 h after infection cells were stained as described for Fig. 4. (B) To assess bacterial killing, infected macrophages with bacteria treated with immune sera were permeabilized with 0.1% saponin and stained using LIVE/DEAD BacLight. SYPRO-9 (green) will enter all bacterial cells, but only membrane-compromised bacteria will integrate PI (red) into DNA, giving a higher red intensity. Panels below each group represent magnifications (10×) of the images on top. Images were taken using an Olympus BX51 upright fluorescence microscope (60×) and processed using Image J software (37). Scale bars, 25 μm.

An essential role for antibodies is dependent on their Fc-associated functionality and includes the ability to promote immunogen uptake. To assess if immunization with AuNP-delivered antigens is associated with this additional role *in vitro*, we infected macrophages with EHEC O157:H7 that was previously incubated in the presence or absence of immune sera. Macrophages infected with EHEC previously treated with immune sera from AuNP vaccine individual formulations demonstrated higher bacterial uptake than naive sera or bacteria alone (Fig. 5A). Furthermore, to assess if macrophage uptake was associated with bacterial killing, we permeabilized macrophages and stained with a bacterial live/dead (green/red) staining kit. In the presence of immune sera, we observed higher bacterial killing by primary murine macrophages of those internalized bacteria than for naive sera or bacteria alone (Fig. 5B). These results demonstrate the functionality of immune sera from AuNP vaccine-immunized animals and their ability to promote macrophage uptake and killing.

## DISCUSSION

Recent vaccine technologies have exploited the use of synthetic materials to achieve enhanced protection against complex pathogens, as was recently described against the severe acute respiratory syndrome coronavirus 2 (SARS-CoV-2) (33). The success achieved by these vaccines is owed, in part, to their tunable physicochemical characteristics like their size and charge, allowing for increased antigen loading and targeted cargo delivery. Gold nanoparticles have been exploited for vaccine design in several diseases using small-animal models of infection (29, 30). However, the skepticism regarding their safety and scaling for mass production, among other hurdles, has considerably set back their development. Nonetheless, the safety observed in our previous studies using AuNPs, together with the ability to induce robust protective responses *in vivo*, offers an avenue for a safe and effective vaccination approach against E. coli O157:H7 and other pathogenic E. coli strains.

**FIG 6:**
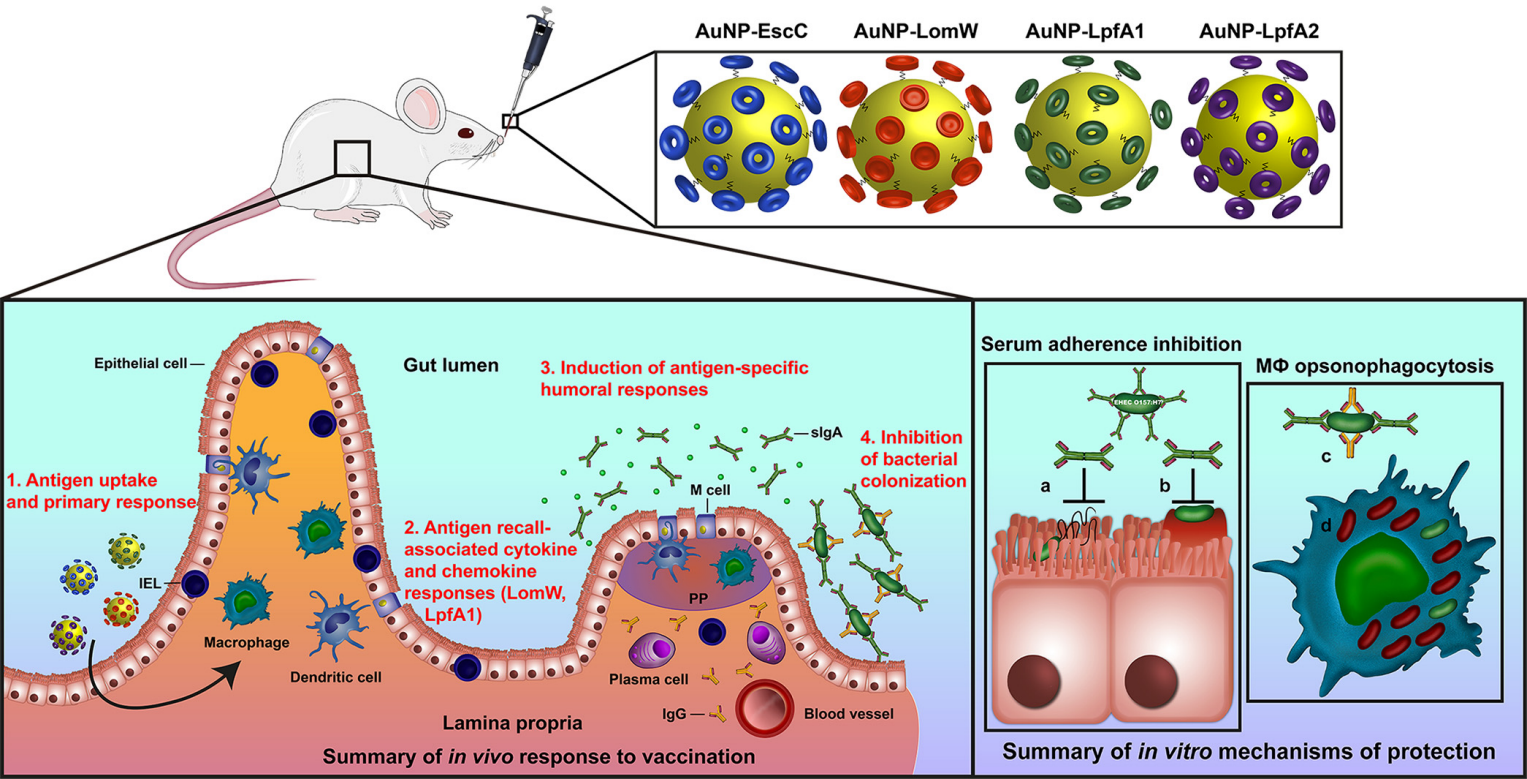
Summary illustration of vaccine protective mechanisms. Intranasal immunization with individual AuNP-protein formulations involving mucosal uptake induces antigen-specific humoral and cell-mediated responses associated with protection against bacterial adherence *in vivo* and *in vitro*.

Vaccination approaches against enterohemorrhagic E. coli O157:H7 have mainly centered on nonspecific vaccine-prime antigenic approaches, with some experiments including outer membrane vesicles and ghost cells (18, 19). We previously reported the immunogenicity and protection of AuNP-delivered antigens identified using a predictive bio- and immunoinformatics algorithm that predicts linear T- and B-cell epitopes and selection of potential immunogenic antigens for vaccine optimization (17, 23–25). Following these initial studies, we wanted to further refine the selection of these candidates to include important colonization factors associated with *in vivo* pathogenesis, such as the fimbrial proteins LpfA1 and LpfA2 (6, 10, 11). In the present study, we further evaluated the protective response associated with nanoparticle-delivered vaccination. In our previous study, we only included LomW and EscC; however, we now evaluated an intranasal route instead of the systemic subcutaneous route to further explore the idea that a mucosal route of immunization elicits stronger mucosal immunity associated with better protection against infection. Furthermore, we used the intranasal delivery route to stimulate a robust mucosal response with the goal of stimulating mucosal antibody and tissue-specific cell-mediated responses. Using our AuNP-coupled antigens as a specific vaccination formulation, we expect that the gold nanoparticles can cross the epithelial barrier, allowing for more efficient antigen uptake, processing, and subsequent responses (Fig. 6). Our data suggest that the intranasal route of vaccination promotes antigen-specific secretory IgA, systemic IgG, and mucosal-specific lymphocyte recall after vaccination (Fig. 6). However, the recall responses were only observed from intraepithelial lymphocytes restimulated with LomW or LpfA1, which suggests that the cell-mediated responses observed are antigen dependent and associated with the number or affinity of T-cell epitopes within the antigens evaluated. These results were similarly described using an influenza challenge in which vaccination with AuNP-HA (hemagglutinin) and flagellin (FliC)-coupled AuNPs induced robust antigen-specific humoral (IgG and IgA in mucosal surfaces) and cell (antigen-specific IFN-γ secreting and CD4^+^ cell proliferation)-mediated responses (34). In addition, these antigen-specific responses were associated with enhanced protection against lethal influenza challenge (34). In our studies, we further showed that the humoral response elicited by our various candidates was associated with reduced bacterial colonization *in vivo*. The functionality of antibodies was further studied *in vitro* with immune sera, which resulted in strong inhibition of adherence onto mouse primary IECs, suggesting a mechanism of serum-mediated membrane-antigen recognition and inhibition, thereby preventing adherence and subsequent pedestal formation. This desirable response further strengthens the need to test rationally selected antigens for vaccine incorporation against bacteria with complex pathogenesis schemes. Furthermore, our data demonstrated that immune sera recognized the outer membrane of EHEC O157:H7, resulting in neutralization. This further suggested that neutralization is an antigen-specific phenomenon and warrants further investigation to define the functionality of LomW, EscC, and LpfA1/2. To further broaden the spectrum of protection associated with our different vaccine candidates, we explored the mechanism of vaccine-induced protection using primary murine intestinal epithelial cells. Bacteria in the presence of immune sera were recognized and uptake increased by primary macrophages. The Fc-mediated uptake of bacteria by primary macrophages strengthens the hypothesis that antibody functionality elicited by the AuNP vaccine is an antigen-specific effect. To better understand the fate of bacterial uptake by primary macrophages, we analyzed the proportion of live/dead bacteria upon cell membrane permeability. Our observations showing a higher bacterial uptake by macrophages and higher proportion of bacterial cells dead suggest an antigen-specific antibody-mediated death effect upon macrophage uptake. The Fc domain recognition by Fc receptors on macrophages, triggering phagocytosis and bacterial death, is a well-recognized antigen-dependent antibody function and has been extensively reviewed using other pathogens, such as Staphylococcus aureus and *Enterococcus* (35).

Overall, our studies demonstrated that optimization of vaccine dose formulation and intranasal route of delivery is an effective route of immunization that is comparable to the systemic vaccination route that we have previously evaluated (23, 25). We have found that intranasal immunization induced robust tissue-specific and systemic humoral responses as well as cellular responses associated with reduced EHEC colonization. These vaccine-induced antibody responses promote bacterial neutralization associated with reduced adherence onto IECs and increased bacterial uptake and killing by mouse primary macrophages. The overall proposed mechanism of vaccine-induced protection both *in vitro* and *in vivo* is summarized in Fig. 6 and highlights the potential vaccine uptake mechanism, the induction of cellular responses by AuNP-LpfA1 and AuNP-LomW, and humoral protective responses for all vaccine candidates as well as the mechanism of antibody-mediated protection. Future experiments will include combinations of these AuNP vaccines to maximize immune responses as well as protective capacity of the vaccine candidates. However, our current data demonstrate the protective nature of the gold-coupled antigens as vaccine formulations against EHEC O157:H7 and warrant further refinement during preclinical studies and prior to beginning investigation in clinical studies.

## MATERIALS AND METHODS

### Bacterial strain and growth conditions.

The diarrheagenic E. coli strain used in this study was EHEC (O157:H7) strain 86-24, which was routinely grown aerobically at 37°C in Luria-Bertani (LB) broth. Before cell infection, the overnight cultures were activated in Dulbecco’s modified Eagle’s medium (DMEM) without fetal bovine serum (FBS) and without antibiotics. Bacterial cultures were incubated for 2 h at 37°C under static growth.

### Cloning.

EHEC (EDL933) genomic DNA was isolated via a Qiagen DNeasy blood and tissue kit according to the manufacturer’s directions. Sequences encoding LomW (GI:12514345), EscC (GI:12518466), LpfA1 (GI:12518278), and LpfA2 (GI:12518581) were amplified via Phusion polymerase (New England BioLabs) and cloned into a pET30a(+) expression vector using NdeI and XhoI or HindIII-HF (New England BioLabs) restriction sites. The open reading frame encoding each protein was inserted in-frame with a 6×-histidine (His) tag on the C terminus. Ligation, transformation, and expression were performed according to the manufacturer’s directions (pET System; Novagen), with some modifications. Upon confirmation of successful gene insertion via gel electrophoresis and directional sequencing (UTMB Genomics Core), plasmids were transformed into BL21(DE3) competent E. coli (New England BioLabs) via heat shock treatment.

### Protein purification.

To induce protein expression, overnight cultures were diluted 1:20 in 1 liter of LB broth, grown to an optical density at 600 nm between 0.6 and 0.8, and induced with 1 mM final concentration of isopropyl β-d-1-thiogalactopyranoside (IPTG). At 3 h postinduction, cultures were centrifuged (4,000 × *g* for 15 min), and the resulting bacterial pellet was frozen at −20°C. The bacterial pellets were then resuspended in phosphate-buffered saline containing 10% glycerol and 25 mM sucrose with 1-mg/ml final concentration of lysozyme, 0.2% sodium deoxycholate, and a tablet of cOmplete EDTA-free protease inhibitor cocktail (Roche, Germany). This lysate was then sonicated and centrifuged, and the pellet was used for subsequent washes to maximize soluble protein extraction. After spindown, the supernatant was filtered sterilized (0.2 mm). Soluble protein extracts were then bound to Talon nickel columns (GE Healthcare, USA) and washed with a PBS buffer containing 50 mM imidazole. Proteins were eluted from affinity columns by applying a PBS buffer with 10% glycerol, 25 mM sucrose, and 250 mM imidazole. Fractions were collected and pooled before dialyzing overnight at 4°C. The purified proteins and protein standards were subjected to a colorimetric bicinchoninic acid (BCA) assay according to the manufacturer’s protocol and then stored at −80°C until use. For protein visualization, 2 mg of protein was run on SDS-PAGE gel. Protein bands were visualized by staining with Coomassie blue stain (Bio-Rad), or gels were transferred to a nitrocellulose membrane for Western blot analysis. A mouse antihistidine antibody (1:5,000) was incubated overnight at 4°C, and horseradish peroxidase (HRP)-conjugated rabbit anti-mouse IgG was used as a secondary antibody. Protein bands were visualized by adding ECL substrate (ThermoFisher Scientific, USA) and imaged on a film.

### Coupling of protein candidates onto AuNPs.

AuNPs, 15 nm in diameter, were synthesized, conjugated to individual proteins, and characterized as previously described (25–27). Briefly, 1 mM gold(III) chloride trihydrate underwent a reduction reaction with 90 mM sodium citrate dihydrate. Particle size and shape were established by transmission electron microscopy (TEM). To stabilize the conjugation of soluble antigens onto the AuNP surface, 0.1 mM 16-mercaptohexadecanoic acid (16-MHDA) and 0.1% Triton X-100 were added to AuNPs. After 2 h of incubation, this solution was filtered with centrifugation (EMB Millipore Amicon Ultra-15, 30-kDa molecular size cutoff) and repeated to ensure coverage. To covalently link soluble protein, 20 mg/ml nanoparticles was added in the presence of 4-(4,6-dimethoxy-1,3,5-triazin-2-yl)-4-methylmorpholinium tetrafluoroborate (DMTMM). The reactions were carried out in a 100 mM borate buffer to allow for conjugation. Attachment of 16-MHDA and protein was confirmed by measuring plasmon resonance via UV-Vis spectroscopy as well as by SDS-PAGE.

### Animal studies.

Female, 6- to 8-week-old BALB/c mice were purchased from Jackson Laboratories (Bar Harbor, ME, USA) and maintained at animal biosafety level 2 (ABSL2). Animals were housed in microisolator cages under pathogen-free conditions with food and water *ad libitum* and maintained on a 12-h light cycle. All animal protocols were reviewed and approved by the Institutional Animal Care and Use Committee of the University of Texas Medical Branch (protocol no. 1007037C). To allow adequate acclimation, mice were housed within the animal facility for 1 week prior to experimentation.

### Immunization and challenge study.

To evaluate the protective immunogenicity of LomW, EscC, LpfA1, or LpfA2 coupled to AuNPs (AuNP-LomW, AuNP-EscC, AuNP-LpfA1, or AuNP-LpfA2), anesthetized BALB/c mice (*n* = 10 per group) were intranasally (i.n.) vaccinated three times in 2-week intervals. Animals received either the AuNP-LomW, AuNP-EscC, AuNP-LpfA1, or AuNP-LpfA2 formulation. Each vaccine formulation contained 5 μg of protein along with 10 μg of detoxified cholera toxin (CT) (Sigma). Control groups received equivalent amounts of adjuvant alone. To evaluate antibody titers, blood was drawn retroorbitally 2 weeks following the last boost. To isolate sera, blood was incubated for 30 min at room temperature to allow for clotting and centrifuged (10,000 × g for 10 min). Serum was removed and stored at −80°C until use. For assays requiring serum, the sera from all immunized animals (*n* = 10) were pooled and stored.

### Infection and bacterial colonization.

Two weeks after administering the last immunization, all mice were challenged with a dose of ∼9 × 10^9^ CFU of E. coli O157:H7 strain 86-24 via gavage (200 μl). Food was restricted 12 h before infection but was administered *ad libitum* throughout the remainder of the study. Two hours prior to the challenge, mice were injected intraperitoneally with cimetidine (50 mg/kg; Sigma) to reduce stomach acidity. To enumerate bacterial colonization in the gastrointestinal tract, mice were euthanized and ceca, large intestines, and small intestines were removed. Sections from the gastrointestinal (GI) tract were homogenized in 1 ml PBS, serially diluted, and plated on MacConkey agar and incubated at 37°C to enumerate bacterial colonization.

### Detection of protein-specific antibodies.

Baseline serum and fecal samples from adjuvant-only, AuNP-LomW-, AuNP-EscC-, AuNP-LpfA1-, or AuNP-LpfA2-vaccinated mice were collected 7 days before prime (day 0) and 14 days after the second boost. Whole blood was collected via retroorbital bleeding and stored in microvette tubes without anticoagulant. The serum was separated by centrifugation and stored at −80°C until use. Intestinal lavage was collected 14 days after the last immunization by washing the small intestine with 1 ml of PBS using a 3-gauge needle and centrifuged to remove fecal debris, and the supernatants were stored at −80°C until use. The protein-specific total IgG and IgA titers were determined by indirect enzyme-linked immunosorbent assay. A microplate (Costar, Cambridge, MA) was coated at 4°C overnight with 1 μg/well of each protein (LomW, EscC, LpfA1, LpfA2) in 1× Dulbecco’s phosphate-buffered saline (DPBS) (Corning, USA). Wells were washed twice with washing buffer (0.05% Tween 20 in 1× DPBS) and then blocked with blocking buffer (0.05% Tween 20, 2% BSA, 1× DPBS) at room temperature (RT) for 2 h. Blocked wells were washed twice before adding sample diluent (1% BSA, 0.05% Tween 20 in 1× DPBS). The sera or intestinal lavage were added to each top dilution well in triplicate, and 2-fold dilutions were performed following incubation at RT for 2 h. Diluted goat anti-mouse IgG or IgA (Southern Biotech, USA) (1:5,000) was added into each well and then incubated for 3 h after washing. Plates were washed four times prior to addition of tetramethylbenzidine (TMB) substrate solution (Invitrogen, USA). The stop solution (2N H_2_SO_4_) was added, and the samples were immediately read at 450 and 570 nm using a microplate reader (BioTek, USA). The results were reported as the reciprocal of the highest titer giving an optical density (OD) reading of at least the means ± 2 standard deviations from baseline sera or intestinal lavage. All assays were performed in triplicate, and results were shown as the mean reciprocal endpoint titer.

### Cell-mediated response from intraepithelial lymphocytes.

Small intestine epithelial cells from AuNP-LomW-, AuNP-EscC-, AuNP-LpfA1-, or AuNP-LpfA2-immunized mice were obtained 2 weeks after the last immunization. IEL were isolated from the small intestine from control (adjuvant-only) and different formulation-vaccinated mice according to an established protocol (36). Single-cell suspensions of isolated IELs were cultured in 12-well cell culture-grade plates (Corning, USA) in duplicate at 1 × 10^6^ cells/ml in RPMI 1640 (Gibco, Life Technologies) supplemented with 10% fetal calf serum (Invitrogen Life Technologies), 1 mM sodium pyruvate, 2 mM l-glutamine, 100 U penicillin/ml, and 100 mg streptomycin/ml (complete medium), with protein antigens as stimuli. The IEL cell suspensions were stimulated with different stimuli for 5 days, including LomW (10 μg/ml), EscC (10 μg/ml), LpfA1 (10 μg/ml), LpfA2 (10 μg/ml), and αCD3/αCD28 magnetic antibody-coupled beads with 30 U/ml mouse recombinant IL-2 and complete medium alone. After 5 days of incubation at 37°C in a humidified atmosphere (5% CO_2_ and 95% air), cell culture supernatants were collected and immediately stored at −80°C until further analysis. Cytokine production was analyzed using a BioPlex kit (Bio-Rad, USA) according to the manufacturer’s instructions. Cytokine analysis of restimulated mouse IEL cell supernatants was done using BioPlex kit (Bio-Rad, USA) analysis according to the manufacturer’s instructions.

### Macrophage survival assay.

C57BL/6 murine bone marrow-derived primary macrophages (BMDM) (no. C57-6030; Cell Biologics Inc., Chicago, IL) were routinely grown in complete primary cell culture medium by following the manufacturer’s instructions (no. M3368; Cell Biologics). Cells were incubated at 37°C and 5% CO_2_. For infection and microscopic analysis, 5 × 10^5^ cells/well were grown in 12-well cell culture-grade plates in round coverslips and incubated overnight prior to treatment. Bacterial inoculum used at a multiplicity of infection of 10 (5 × 10^6^ CFU) was incubated in the presence or absence of immune serum from immune groups (AuNP-LomW, AuNP-EscC, AuNP-LpfA1, and AuNP-LpfA2) or naive sera (final concentration of 10%) for 1 h at 37°C, with slight agitation. After incubation in the presence or absence of sera, bacteria were collected in 1 ml of fresh medium and used to infect cell culture plates containing 5 × 10^5^ cells. After 2 h of infection at 37°C with 5% CO_2_, cells were washed and fixed with 4% paraformaldehyde–PBS for 30 min. Following that step, cells were slightly permeabilized with 0.1% saponin in PBS for 10 min at room temperature. Cells were then stained with a LIVE/DEAD BacLight (Molecular Probes, Life Technologies) containing propidium iodide (PI) or SYTO 9 by following the manufacturer’s instructions. Cells were washed three times with PBS, fixed with 4% paraformaldehyde for 20 min, and then directly mounted using ProLong gold antifade (Molecular Probes, Life Technologies). Cells were visualized using an Olympus BX51 upright fluorescence microscope and analyzed using ImageJ software from the National Institutes of Health (37).

### Serum-mediated bacterial adherence inhibition assay.

C57BL/6 mouse primary small intestinal epithelial cells (catalog no. C57-6051; Cell Biologics Inc., Chicago, IL) were routinely grown in complete primary cell culture medium by following the manufacturer’s instructions (catalog no. M6621; Cell Biologics Inc.). For adhesion assays, 12-well plates were seeded with 1 × 10^6^ cells/per well. Approximately 1 h prior to infection, the monolayer was washed twice with 1 ml PBS prior to addition of 1 ml medium containing no supplements. Overnight cultures of EHEC O157:H7 strain 86-24 were diluted in DMEM (1:20) without supplements and incubated at 37°C under static growth conditions for 2 h. Bacterial inoculum was adjusted to a multiplicity of infection (MOI) of 10 (1 × 10^7^ CFU) and incubated in the presence of AuNP-LomW, AuNP-EscC, AuNP-LpfA1, or AuNP-LpfA2 immune serum, adjuvant-only serum, or naive serum (10%) for 1 h at 37°C with slight agitation. After incubation in the presence of sera, bacteria were collected in 1 ml of fresh medium and used to infect cell culture plates containing 1 × 10^6^ cells. Monolayers were incubated for 2 h at 37°C with 5% CO_2_. After incubation, cells were washed three times with PBS prior to addition of 100 μl of 0.1% Triton X-100 in PBS. After detachment, the cells were serially diluted in PBS and plated on LB agar plates to enumerate adhered bacteria (output). The percentage of adhered bacteria was determined as the output/input × 100. Data were representative of two independent experiments using pooled sera from *n* = 10 mice. For microscopy analysis, primary intestinal epithelial cells were cultured in round coverslips placed at the bottom of 12-well plates and incubated for 24 h at 37°C with 5% CO_2_. Bacterial inoculum using an MOI of 10 (1 × 10^6^ CFU) was incubated in the presence of AuNP-LomW, AuNP-EscC, AuNP-LpfA1, AuNP-LpfA2, or naive sera (10%) for 1 h at 37°C with slight agitation. After incubation in the presence or absence of sera, bacteria were collected in 1 ml of fresh medium and used to infect cell culture plates containing 1 × 10^5^ cells. Additionally, evaluation of bacterial viability was performed by using the LIVE/DEAD staining (LIVE/DEAD *Bac*Light bacterial viability kit; Invitrogen, USA) by using the manufacturer’s instructions. For cell infection, monolayers were incubated for an additional 2 h at 37°C with 5% CO_2_. After infection, cells were washed and fixed with 4% paraformaldehyde-PBS.

### Fluorescence microscopy and bacterial serum recognition.

For fluorescence microscopy analysis, fixed and permeabilized primary intestinal epithelial cells or primary macrophages were used. Polymerized actin was detected by staining with tetramethyl rhodamine isothiocyanate-phalloidin (Molecular Probes-Invitrogen, USA). DNA from cell nuclei and bacteria were mounted using Fluoroshield and detected with 4′,6-diamidino-2-phenylindole (DAPI). EHEC (86-24) was detected by immunofluorescence with anti-E. coli O plus E. coli K antibody coupled to fluorescein isothiocyanate (FITC) (Abcam, USA). Evaluation of bacteria serum recognition was performed by interaction of EHEC O157:H7 strain 86-24 with sera (1:500) from different immune groups (AuNP-LomW, AuNP-EscC, AuNP-LpfA1, or AuNP-LpfA2) during 1 h at 37°C while shaking. After incubation, cells were washed, fixed, and stained with a goat anti-mouse conjugated to Alexa-488 secondary antibody (1:10,000). Bacterial DNA was visualized using DAPI (Molecular Probes, Invitrogen) and mounted using ProLong Gold antifade (Molecular Probes, Invitrogen). Images were taken using an Olympus BX51 upright fluorescence microscope and analyzed by Image J software (37).

### Statistical analysis.

All statistical analysis was done using GraphPad Prism software (v8.0). *P* values of <0.05 were considered statistically significant. Quantitative data are expressed as the means ± standard errors. All data were analyzed for normality before running the corresponding test. Colonization, antibody results, serum adherence inhibition, and bactericidal assays were analyzed by one-way analysis of variance (ANOVA) (Tukey posttest) or Kruskal-Wallis when data were not normally distributed.
