# Supplementary figures and images for: Optimization of Multivalent Gold Nanoparticle Vaccines Eliciting Humoral and Cellular Immunity in an In Vivo Model of Enterohemorrhagic Escherichia coli O157:H7 Colonization
Source: mSphere. 2022 Jan 19;7(1):e00934-21. doi: 10.1128/msphere.00934-21 (PMC8769200; doi:10.1128/msphere.00934-21)

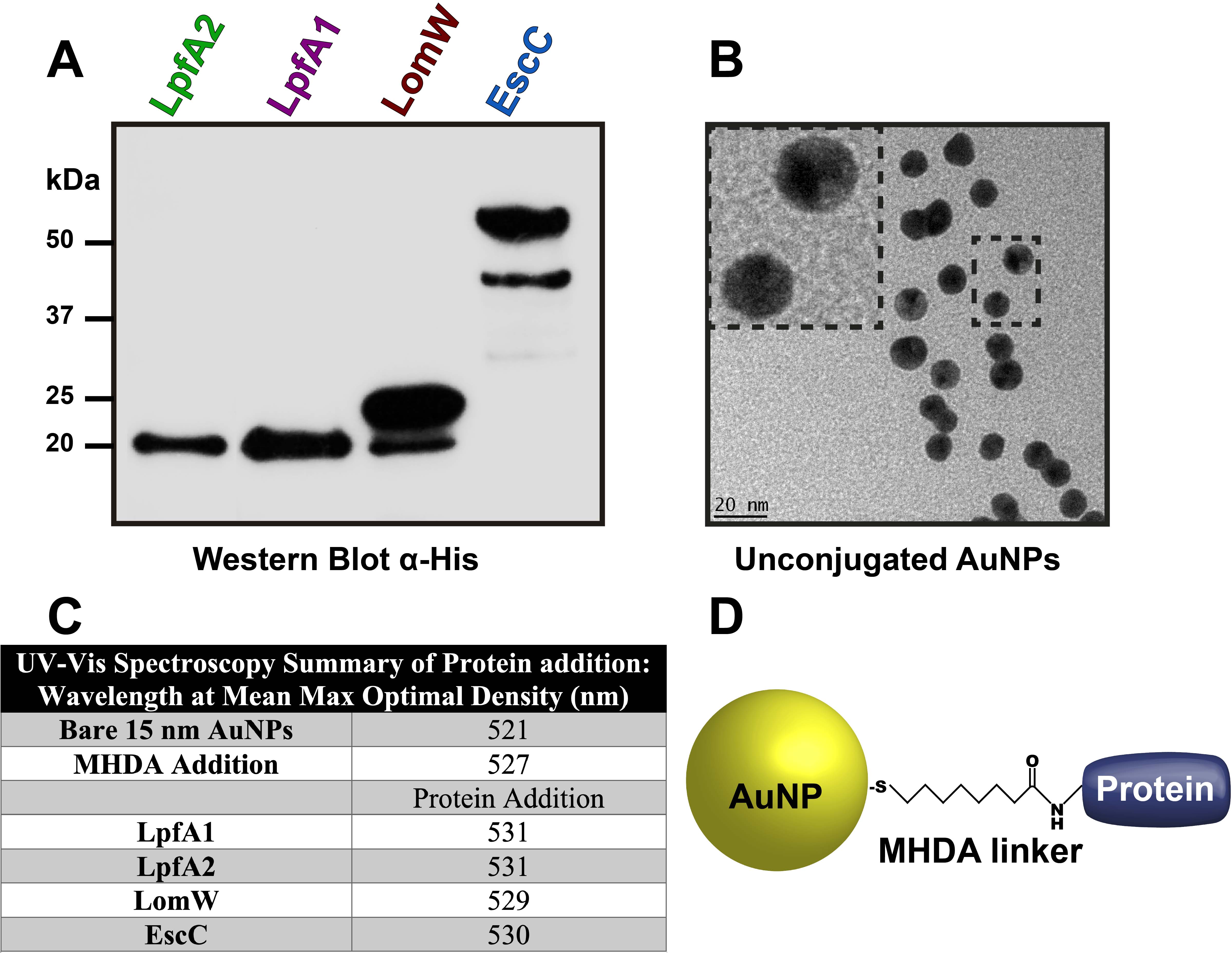

Supplement: FIG S1 [file msphere.00934-21-sf001.tif]
